# The stem cell regulator PEDF is dispensable for maintenance and function of hematopoietic stem cells

**DOI:** 10.1038/s41598-017-09452-2

**Published:** 2017-08-31

**Authors:** Emma Rörby, Matilda Billing, Maria Dahl, Sarah Warsi, Silja Andradottir, Kenichi Miharada, Kavitha Siva, Jan-Ingvar Jönsson, Ulrika Blank, Göran Karlsson, Stefan Karlsson

**Affiliations:** 1 Department of Molecular Medicine and Gene Therapy, Lund Stem Cell Center, Lund University Hospital, 22184 Lund, Sweden; 2 Department of Clinical and Experimental Medicine, Linköping University, 58185 Linköping, Sweden; 3 Division of Molecular Hematology, Lund Stem Cell Center, Lund University, 22184 Lund, Sweden

## Abstract

Pigment epithelium derived factor (PEDF), a ubiquitously expressed 50 kDa secreted glycoprotein, was recently discovered to regulate self-renewal of neural stem cells and have a supportive effect on human embryonic stem cell growth. Here, we analyzed expression of PEDF in the murine hematopoietic stem cell (HSC) compartments and found that PEDF is highly expressed in primary long-term HSCs. Therefore, we characterized the hematopoietic system in a knockout mouse model for PEDF and using this model we surprisingly found that PEDF is dispensable for HSC regulation. PEDF knockout mice exhibit normal hematopoiesis in steady state conditions and the absence of PEDF lead to normal regeneration capacity in a serial competitive transplantation setting. Additionally, PEDF-deficient cells exhibit unaltered lineage distribution upon serial transplantations. When human cord blood stem and progenitor cells were cultured in media supplemented with recombinant PEDF they did not show changes in growth potential. Taken together, we report that PEDF is not a critical regulatory factor for HSC function during regeneration *in vivo* or growth of human stem/progenitor cells *in vitro*.

## Introduction

Hematopoiesis, the process of blood cell formation, relies on a rare population of multipotent hematopoietic stem cells (HSCs). These are primitive, tissue-specific somatic stem cells that can differentiate along all lineages of the hematopoietic system and simultaneously self-renew. Because of this, HSCs have tremendous therapeutic potential and have been used in the clinic for cell and gene therapy of hematopoietic disorders^1^. Expansion of HSCs and progenitor cells *ex vivo* is expected to be highly beneficial and of great clinical relevance making HSCs from cord blood (CB) assessable for adult patients in need^2^. However, expansion of HSCs *in vitro* has met limited success due to incomplete knowledge about how HSCs are controlled. Regulation of HSC fate options by intrinsic and extrinsic factors determines whether HSCs will self-renew, differentiate or undergo apoptosis^1–3^. Improved engraftment after *ex vivo* culture can be obtained through increased self-renewal, improved homing or prolonged survival. Preferably, not yet identified secreted factors controlling HSCs would be of great use to improve expansion culture conditions. To be able to control cell fate in future *in vitro* protocols it is critical to understand how the HSCs are regulated in their natural environment.

Here, we show for the first time using a robust knockout model that the well-known stem cell regulator Pigment epithelium-derived factor (PEDF) does not regulate HSCs despite its critical role for self-renewal of various other tissue types^4–8^.

PEDF is a 50 kDa secreted glycoprotein, encoded by the *Serpinf1* gene, that belongs to the superfamily of serpin protease inhibitor proteins, but lacks inhibitory function^9^. PEDF protein was first purified from the conditioned media of human retinal pigment epithelial cells and has been attributed potent inhibitory functions in physiological and pathological angiogenesis^10–12^. Several lines of evidence suggest that PEDF is an important regulatory factor for self-renewal and differentiation^6–8, 13, 14^. For example, PEDF is among the proteins that have been identified in mesenchymal stem cell-conditioned media^15^ and Gonzalez *et al*. identified PEDF in a screen to search for secreted factors involved in human embryonic stem cells (hESC) maintenance^6^. Feeder cells lose their supportive capacity following long-term culture *in vitro* and Anisimov *et al*. reported that PEDF is highly expressed in fibroblast during early passages and lose expression in intermediate and late passages^14^. Based on these findings PEDF is thought to play am important role in the support of hESC growth. In another study PEDF was infused into the lateral ventricle of adult mice, which increased the number of self-renewing neural stem cells *in vivo* ^4^. Interestingly, PEDF was one of 62 genes previously identified by gene expression profile analysis as downregulated when a pluripotent, self-renewing hematopoietic cell line entered differentiation^16^. We analyzed expression of PEDF by real time–PCR and in agreement with the previously published cell line data we found that PEDF is highly expressed in primary long-term HSCs. Therefore, we investigated the role of PEDF in the regulation of hematopoiesis *in vivo* during steady state and regeneration. Surprisingly, we observed that PEDF is not required for normal repopulation capacity. Loss of PEDF in adult bone marrow (BM) cells resulted in normal hematopoiesis in steady state mice and when investigating stressed hematopoiesis during competitive transplantation we found no change in repopulation capacity of PEDF-deficient cells. Furthermore, the absence of PEDF did not change the engraftment or lineage distribution upon serial transplantation. PEDF has been shown to have important roles in several stem cell culture systems including embryonic, retinal and mesenchymal stem cell cultures^6, 7, 13, 14, 17^. However, PEDF did not affect CB hematopoietic stem and progenitor cell (HSPC) growth *in vitro*.

Taken together, the results of the present study can unexpectedly rule out an essential role for PEDF in the hematopoietic system.

## Results

### PEDF is highly expressed in wild type HSCs but steady state PEDF null mice exhibit normal hematopoiesis

**Figure 1:**
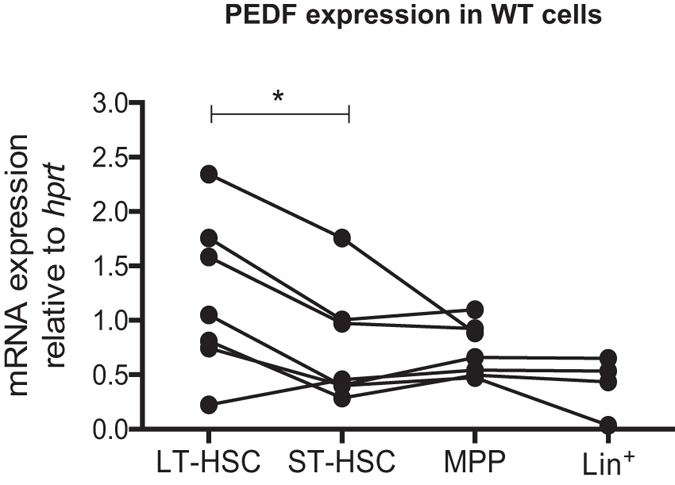
PEDF is highly expressed in HSCs. Wild type cells were sorted for LSKFlt3^−^CD34^−^ (LT-HSC), LSKFlt3^−^CD34^+^ (ST-HSC), LSKFlt3^+^CD34^+^ (MPP) and Lineage positive (Lin^+^) cells and PEDF mRNA expression was measured by qPCR. Line shows increase/decrease in PEDF expression between the populations for each independent experiment (n = 7, *P* = 0.0101 paired Student t test).

**Figure 2:**
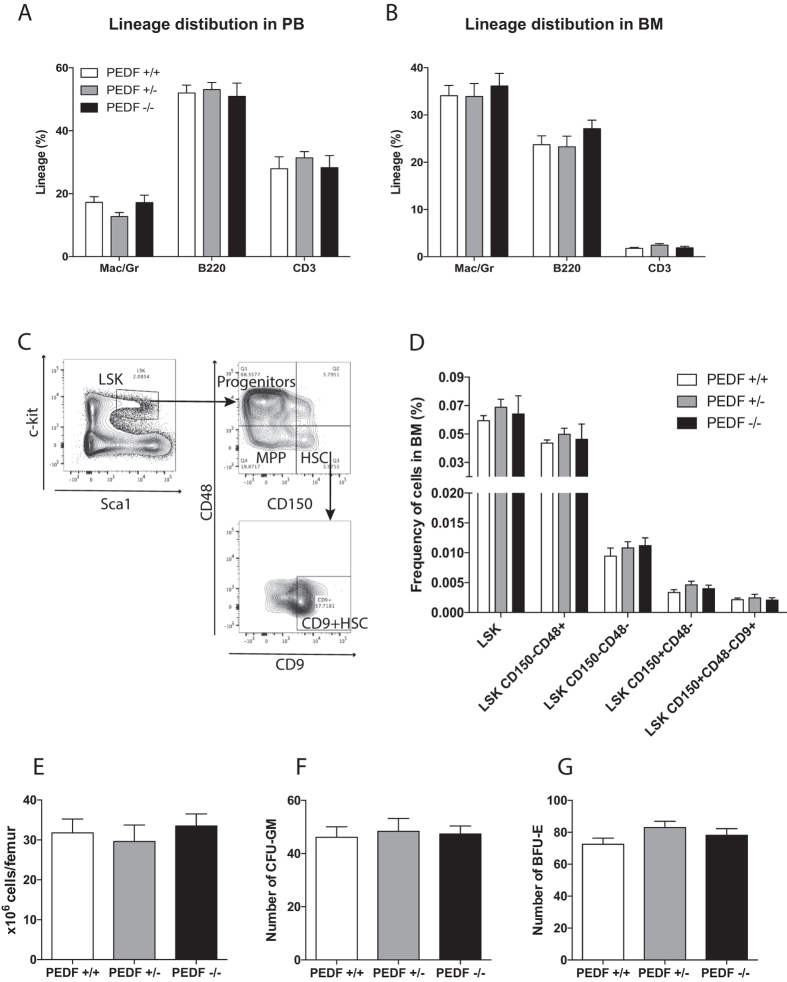
PEDF−/− steady state BM had normal hematopoiesis. Flow cytometric analysis of lineage distribution in PB (**A**) and BM (**B**) of steady state mice. Representative FACS plots of stem cell analysis (**C**) and the graph represents frequencies of stem and progenitor cells in BM of steady state mice (**D**), HSCs (LSKCD150^+^CD48^−^CD9^+^, LSKCD150^+^CD48^−^), MPPs (LSKCD150^−^CD48^−^) and progenitor cells (LSKCD150^−^CD48^+^, LSK). Number of cells per femur (**E**) and colony count for colony forming unit granulocyte-monocyte, CFU-GM (**F**) and burst forming unit-erythroid, BFU-E (**G**) from BM cells plated in methylcellulose media. Data are mean ± SEM, n = 4.

Genes highly expressed in HSCs compared to progenitors and mature cells may play an important role in fine-tuning HSC properties and fate. Interestingly, PEDF have been shown to be expressed at highest levels in murine HSCs (https://gexc.riken.jp/models/3/genes/Serpinf1?q=serpinf1) based on The Gene Expression Commons, Stanford University in which microarray expression profiles of immature and mature hematopoietic subsets can be visualized^18^. Furthermore, another study showed that PEDF expression levels in FDCP-mix cells are downregulated upon differentiation^19^. Together, these results indicate that PEDF expression is important in primitive HSCs and that it declines as HSCs undergo differentiation into mature blood lineages. To confirm the high expression level in primary hematopoietic cells we performed mRNA analysis of the blood stem cell compartments and found that PEDF is highly expressed in long-term (LT)-HSCs and present at lower levels in short term (ST)-HSCs, multipotent progenitor cells (MPPs) and more mature lineage positive cells (Lin^+^) (Fig. 1). By FACS analysis we observe that approximately ten percent of HSCs (LSKCD150 + CD48−) express the PEDF receptor (PEDFR) (Supplementary Figure 1A). This prompted us to investigate a potential role for PEDF in HSCs by analyzing HSC function in a non-inducible PEDF−/− transgenic mice model. PEDF−/− mice, on a C57BL/6 J × 129S6 background, had been generated using a novel knock-in methodology where the whole *Serpinf1* gene was replaced with a targeted vector encoding a lacZ reporter cassette^20^. PEDF−/− mice were backcrossed for 11 generations using C57BL/6 J wild type mice. PEDF-deficient mice appeared healthy and exhibited no overt developmental phenotype and we confirmed efficient knockout of PEDF in primitive HSCs (LSKCD150 + CD48−) (Supplementary Figure 1B). To gauge the impact of PEDF in steady state mice we performed detailed immunophenotyping and differential blood counts of mature hematopoietic lineages. To determine if a specific lineage might be affected in the PEDF-deficient mice we analyzed lineage distribution in peripheral blood (PB) and BM, but no change was observed compared to littermate controls (Fig. 2A and B). Moreover, bone morphology of PEDF-deficient mice revealed no change in bone marrow histopathology (data not shown).

Furthermore, we examined complete blood counts and found that the blood samples did not differ in white blood counts, platelets, hemoglobin, or red blood cell levels in PEDF-deficient mice compared to controls (data not shown). We then used flow cytometry analysis to estimate the frequencies of the most primitive HSCs (LSKCD150+CD48−CD9+)^21^, MPPs (LSKCD150−CD48−), and progenitor cells (LSKCD150−CD48+, LSK), but we could not observe any difference in the proportion of the various stem and progenitor cell populations between littermate controls and PEDF-deficient animals (Fig. 2C and D). Additionally, BM cell numbers were equal in all groups (Fig. 2E).

Next, we wanted to examine if the loss of PEDF would affect cell proliferation *in vitro*. We asked whether PEDF expression was important for the differentiation of hematopoietic progenitor cells using methylcellulose cultures to measure colony capacity. However, we found no differences in number of colonies or colony size between PEDF knockout cells and control cells (Fig. 2F and G).

Taken together, despite the high expression levels in primitive wild type cells we conclude that PEDF null mice have normal hematopoietic status during steady state conditions.

### PEDF is dispensable for regulation of HSC survival and maintenance after BM transplantation

**Figure 3:**
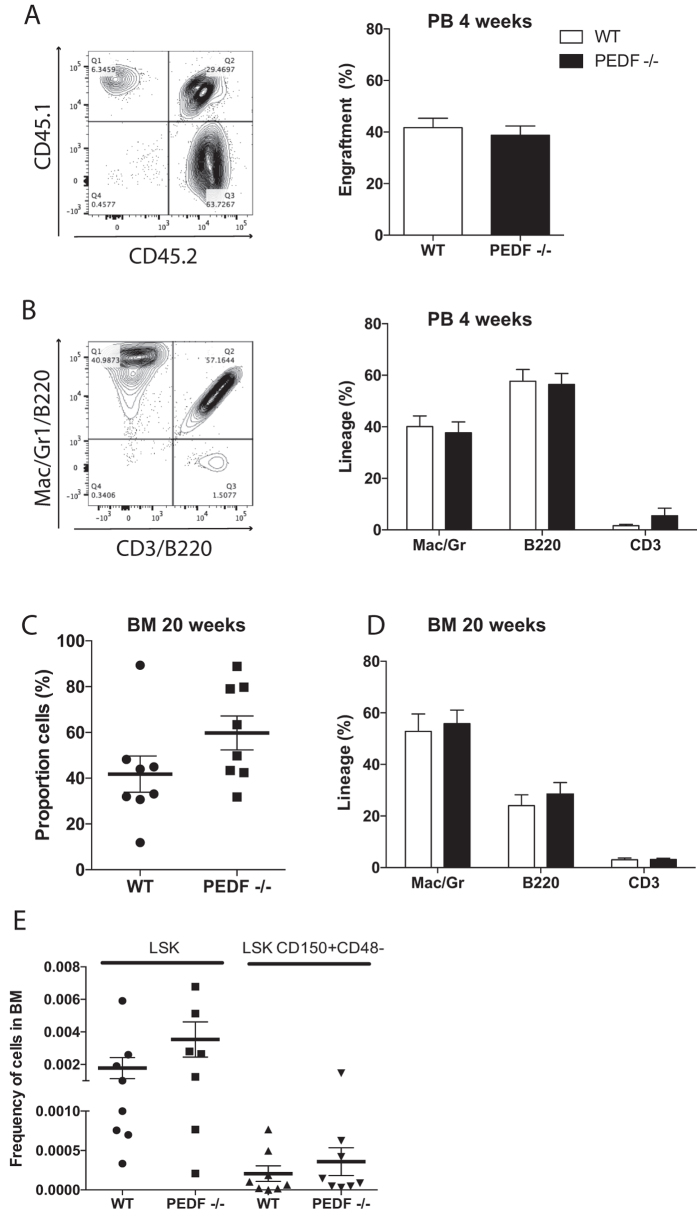
PEDF−/− BM cells give rise to stable reconstitution and normal hematopoiesis in competitive transplantation experiments. (**A**) Engraftment in primary recipients measured by flow cytometric analysis of engrafted CD45.2^+^ donor cells in recipient PB at four weeks. (**B**) Lineage distribution of CD45.2^+^ cells in PB at four weeks time point, myeloid cells: Mac/Gr, B-cells: B220 and T-cells: CD3. (**C**) Engraftment in BM of primary transplanted recipients 20 weeks post transplantation. (**D**) Lineage distribution 20 weeks post transplantation in BM of primary transplanted mice. (**E**) Flow cytometric analysis of LSKCD48^−^CD150^+^ cell and LSK cell frequencies in BM of primary recipients. Data are mean or shows individual mice (**C** and **E**) from three independent experiments ± SEM, n = 3 donors per genotype and 2–3 recipients per donor.

To determine the effect of PEDF on the *in vivo* function and reconstitution ability of HSCs we performed competitive repopulation assays in which BM cells (CD45.2) from PEDF knockout mice or WT mice were mixed at a 1:1 ratio with WT competitors (CD45.1/CD45.2) and transplanted into lethally irradiated primary recipient mice (CD45.1), engraftment potential and lineage distribution was analyzed post transplantation (Fig. 3A–D). Engraftment (% 5.2 cells) and lineage distribution in PB at four weeks showed no significant differences between WT and PEDF knockout donor cells in neither engraftment of total 5.2+ cells nor the myeloid and lymphoid lineage distribution (Fig. 3A and B), suggesting that PEDF is not needed for HSC homing or short-term reconstitution in primary transplants. Moreover, the proportion of 5.2+ long-term engrafting cells and lineage distribution in BM at 20 weeks after transplantation were normal (Fig. 3C and D) as well as the frequencies of the Lin-Sca1^+^c-Kit^+^ (LSK) stem and progenitor population and highly purified HSCs (LSKCD150^+^CD48^−^) (Fig. 3E).

**Figure 4:**
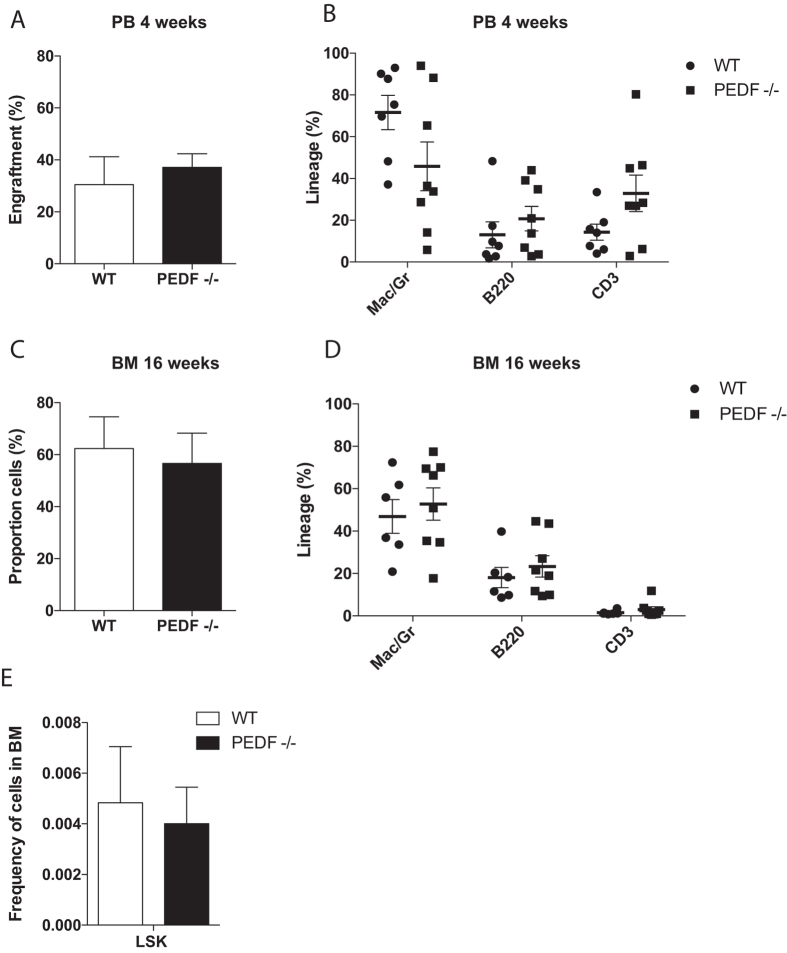
PEDF deficiency had limited effect on long-term hematopoiesis and HSC function in secondary transplantations. (**A**) Engraftment, percent CD45.2 + cells, and lineage distribution (**B**) in PB of secondary recipients 4 weeks post transplant. (**C**) The graphs show engraftment, proportion of CD45.2 cells, in total BM and lineage distribution (**D)** 16 weeks post transplant in BM of secondary transplanted mice. (**E**) Graph shows FACS analysis of LSK cells in BM 16 weeks after secondary transplantation. Results shown are the mean ± SEM from three independent experiments (n = 3 donors per genotype and 2–3 recipients per donor).

To expose HSCs to extreme proliferation stress and to analyze the ability to confer stable hematopoiesis we performed secondary transplantations where primary BM cells were transplanted into lethally irradiated secondary recipient mice and analyzed at 4 weeks post transplantation (Fig. 4A and B). PEDF-deficient recipient mice showed no disadvantages in frequencies of donor cells compared to WT controls or lineage distribution at this time point. Also, four months after secondary transplantation we found normal reconstitution as compared to WT donor cells (Fig. 4C and D), clearly showing that PEDF-deficient HSCs have similar functional potential compared to WT control HSCs. We also examined the LSK frequency of secondary recipient mice after harvesting their BM and saw no significant differences between the two groups (Fig. 4E).

Taken together, these results establish that PEDF does not have an essential role in the maintenance of the HSC compartment in adult mice.

### PEDF does not influence human HSPC growth potential *in vitro*

**Figure 5:**
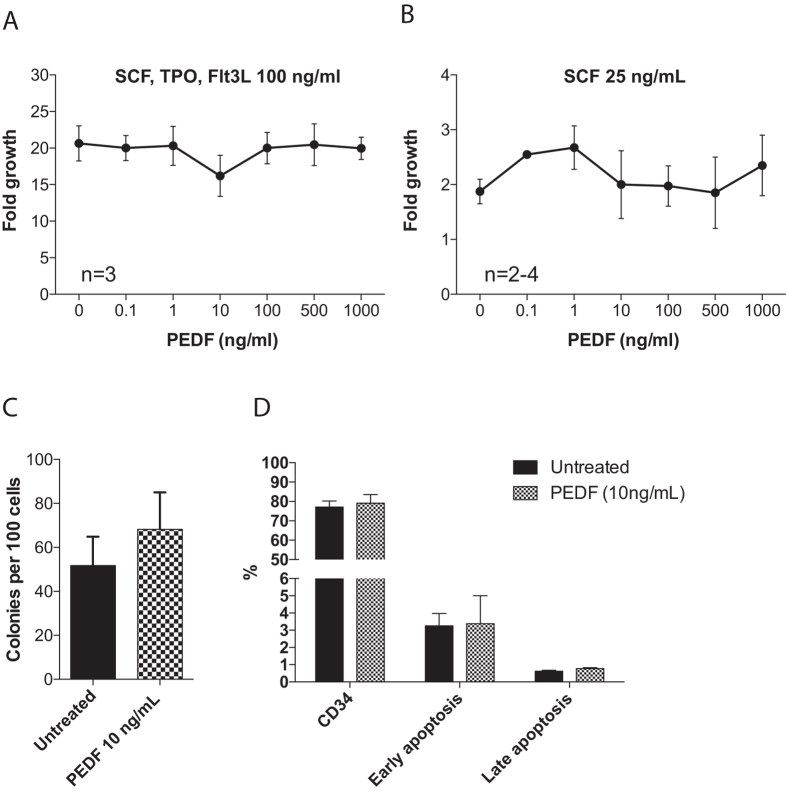
Dose response curve for recombinant human PEDF in human HSPCs. PEDF-dose response curve for CD34 + cord blood cells. (**A**) Cells were cultured in SFEM media supplemented with SCF, TPO and Flt3L, all cytokines in 100 ng/ml with or without recombinant human PEDF at given concentrations (n = 3). (**B**) CD34 + cells were cultured in SFEM supplemented with 25 ng/ml SCF with or without recombinant human PEDF at given concentrations (n = 2–4). (**C**) After five days of culture with or without recombinant PEDF in SFEM media (supplemented with SCF, TPO and Flt3L) cells were plated in methylcellulose media. Graph shows number of colonies per 100 cells plated (n = 4). (**D**) Graph shows percentage of cells expressing CD34 or apoptotic markers after five days of culture in SFEM (supplemented with SCF, TPO and Flt3L) (n = 2). Results shown are the mean ± SEM.

PEDF has important growth supportive effects in other stem cell systems^5–7, 13, 14^. For example, it has been shown that PEDF promotes proliferation and long-term growth of pluripotent human embryonic stem cells^5, 6, 14^. Even tough, human hematopoietic stem cells from CB did not have the highest expression of PEDF (Supplementary Figure 2), which correlates to the Gene Expression Commons database (https://gexc.riken.jp/models/-7/genes/SERPINF1?q=Serpinf1), we next sought to determine the involvement of recombinant PEDF in growth control of human cord blood HSPCs in *in vitro* culture. Given the fact that PEDF has been associated with a G_0_ quiescent state of cell cycle in fibroblasts and that previous studies have seen an increased self-renewal potential of neural stem cells after PEDF administration^4, 22^, we sought to determine whether adding recombinant PEDF to the culture media could alter growth either in an inhibitory or stimulatory way. We used two culture conditions, either SFEM medium supplemented with three cytokines; SCF, TPO and Flt3L all at a concentration of  100 ng/ml, and a second low stimulatory culture condition only adding a small amount of SCF (25 ng/ml) to SFEM medium. However, in both culture conditions we did only see a modest effect of PEDF in a concentration range between 0.1–1000 ng/ml (Fig. 5A,B). After 5 days of culture in media supplemented with SCF, TPO and Flt3L with or without PEDF (10 ng/ml) we seeded out HSPCs into methylcellulose cultures. However, both the colony number and colony size were comparable between the two preconditions, indicating that PEDF cannot affect symmetric self-renewal or inhibit stem cell growth in our culture condition (Fig. 5C). As expected, we confirmed that the primitive CD34^+^ population did not change in frequency after 5 days of culture and that apoptosis was equal between the groups (Fig. 5D).

## Discussion

To improve future *in vitro* protocols for HSC expansion it is important to get deeper understanding of molecular factors controlling HSC fate decisions such as self-renewal and differentiation. PEDF has been shown to be involved in regulating growth of neural stem cells, retinal stem cells and human embryonic stem cells^4, 6–8^, and is thus a potential growth factor to be used for better expansion conditions of HSCs. Interestingly, PEDF is generally regarded as a secreted protein but several immunohistochemical studies have reported significant quantities of PEDF in the nucleus of different cell types including retinal pigment epithelial cells, Y-79 retinoblastoma cells, NA neuroblastoma cells and hepatocarcinoma HepG2 cells^23–26^. Furthermore, in a recent study PEDF was shown to bind importin-b family member transportin-SR2, which is associated with active nuclear transport^27^. These findings suggest that PEDF might not only work as a secreted factor but could also have cell-intrinsic gene regulatory functions. In an effort to decipher the role of PEDF in hematopoietic stem cells we have studied a knockout mouse model for PEDF.

To our surprise we found that PEDF is dispensable for the maintenance and differentiation of HSCs even though PEDF was highly expressed in wild type LT-HSCs and expressed at a lower level in ST-HSCs and progenitor cells in our mRNA analysis. Based on the assays performed here, steady state knockout mice have a normal phenotype, blood count and colony forming capacity. Our observations show that loss of cell-autonomous PEDF failed to demonstrate a significant effect on HSCs. Interestingly, PEDF is expressed in most body tissues and many cell types have been shown to express and secrete PEDF including osteoblasts, mesenchymal cells and Schwann cells^28–31^. Importantly, our model is a complete non-inducible knockout model illustrating that systemic deletion of PEDF does not affect HSC function. Since Sun *et al*. have shown that long-lived progenitors rather than the stem cells are the main drivers of hematopoiesis during steady state conditions, we wanted to challenge the system by performing a transplantation to test the stem cell function in PEDF-deleted cells^32^. To determine how PEDF−/− HSCs function in a highly proliferative environment we competitively transplanted WT or PEDF-deficient cells into lethally irradiated recipient mice. PEDF-deficient hematopoietic cells, showed no disadvantages in transplantation experiments with equal frequencies of engraftment and lineage distribution at both short-term (four weeks) and long-term (20 weeks) time points. Interestingly, Gattu *et al*. have shown that PEDF can modulate differentiation in other contexts^33^, but here we show that the myeloid and lymphoid lineage distribution of recipient mice was normal. To expose HSCs to extreme stress having to rebuild the hematopoietic system in secondary recipients we found that PEDF-deficient donor cells did not exhibit any phenotypical or functional abnormalities in their HSC compartment even at this setting giving rise to stable long-term hematopoiesis also in secondary recipient mice. It is possible that PEDF is not important for adult hematopoiesis but serve a role in fetal HSC function. Although, future work is required to identify the role of PEDF in fetal hematopoiesis, our data clearly demonstrate that PEDF is dispensable for adult HSC function.

Accumulating evidence demonstrates a role for PEDF in a variety of stem cell culturing systems. PEDF has been shown to support stem cell survival and maintaining multipotency^5, 6, 14^. An example is that PEDF is highly expressed in fibroblasts that serve as feeder cells supporting self-renewal of human embryonic stem cells^6, 14^. Moreover, proliferation of limbal epithelial stem cells, stem cells in the eye, has recently been shown to be enhanced by PEDF treatment *in vitro* ^5^. In our hands, recombinant human PEDF could not influence growth potential of human HSPCs from cord blood. *In vitro* culturing of HSPCs in two different media conditions did not change the proliferation rate or the colony forming capacity (Fig. 5). In agreement with this, we did not see any changes in the frequency of apoptotic cells or primitive CD34^+^ population of cells that had been cultured with or without PEDF. It is possible that the concentration of recombinant PEDF is not within an optimal range, although we evaluated six different concentrations (ranging from 0.1 to 1000 ng/ml) of PEDF. However, our *in vitro* data from cultured CB cells is consistent with our knockout model were systemic deletion of physiological PEDF expression does not seem to affect stem cell function *in vivo*.

It should be emphasized that the model systems reported before that suggest a role for PEDF as a stem cell regulator are different than the model used here. PEDF was reported to be a regulator of self-renewal in human ES cells whereas we use PEDF null mice in most of our studies. Similarly, a role for PEDF as a regulator of neural stem cells was tested in mice that are different from the PEDF null mice investigated in this study. Therefore, the difference between the published findings on ES cells and neural stem cells may be due to a species difference, a mouse strain difference or the different results may be due to different effects of PEDF on different types of stem cells.

In conclusion, our results demonstrate that PEDF is not involved in HSC regulation despite expression and previous reports. Loss of PEDF leads to normal repopulation capacity followed by normal myeloid and lymphoid lineage development in murine transplantation settings. Based on the assays performed here, steady state mice have a normal phenotype and colony forming capacity. Thus, PEDF is not crucial for HSCs neither under homeostasis nor regenerative conditions. Furthermore, recombinant human PEDF could not influence the growth and survival of hematopoietic stem/progenitor cells from human cord blood.

## Materials and Methods

### Mice

PEDF−/− mice were bred in the animal facility at the biomedical center, Lund University. Mice were kept in ventilated racks and given autoclaved food and water. Lund University’s ethical committee approved all animal experiments. All experimental methods involving animals were carried out in accordance the relevant guidelines and regulations from Lund University ethical committee for animal research. Mice were provided by Dr. S.J. Wiegand (Regeneron Pharmaseuticals, Tarrytown, NY). PEDF−/− mice, on a C57BL/6J × 129S6 background, had been generated using a novel knock-in methodology where the whole *Serpinf1* gene was replaced with a targeted vector encoding a lacZ reporter cassette^20^. PEDF−/− mice were backcrossed for 11 generations using C57BL/6J wild type mice.

### BM transplantation assay

Bone marrow (BM) was harvested from 7–12 week old PEDF−/− and wild type control mice (CD45.2) and 2 × 10^5^ BM cells were competitively transplanted together with 2 × 10^5^ BM cells from C57BL6xB6SJL (Ly5.1/Ly5.2) into the tail vein of lethally irradiated (900 cGy) C57BL6 recipient mice (Ly5.1). In case of secondary transplantation ½ of a femur was passaged into new lethally irradiated recipients 20 weeks after primary transplantation. Bone marrow from secondary transplanted mice was analyzed 16 weeks post transplantation and peripheral blood (PB) was analyzed at four and 16 weeks post transplantation. Three independent experiments were performed, 9 mice were transplanted for each genotype in total.

### Cell preparations

PB was collected from the tail veins and kept in Microvette tubes (Sarstedt) until further analysis. When necessary, red blood cells were lysed with ammonium chloride (NH_4_Cl; Stem Cell Technologies). PB was analyzed using SYSMEX XE-5000. Recipient mice were euthanized and femur and tibiae were crushed in a mortar to collect BM cells. Cell suspensions were filtered through a 70 μm nylon cell strainer (BD Bioscience) in phosphate-buffered saline (PBS; Invitrogen) containing 2% FCS (Invitrogen) and analyzed by flow cytometry.

### Flow cytometry

Cells from mouse PB, spleen, unfractionated BM or magnetically c-kit enriched BM or human CB cells were stained in PBS (Invitrogen) supplemented with FCS (Invitrogen) together with combinations of antibodies. For full list of antibodies, see supplemental Table 1. Dead cells were detected by staining with 7-aminoacinomycin D (7-AAD; Sigma-Aldrich. Cells were analyzed using FACSCanto (BD Biosciences). For sorting cells a FACSArial cell sorter (BD Biosciences) was used.

### *In vitro* cultures and colony assay of mouse cells

For granulocyte-macrophage colonies 30 000 fresh BM cells from steady state mice were plated in methylcellulose medium (M3231; StemCell Technologies) containing 50 ng/mL mSCF, 10 ng/mL murine interleukin 3 (IL-3; PreproTech), 10 ng/mL human IL-6 (PreproTech), 100 IU penicillin and 100 μg streptomycin (P/S; Invitrogen) in 35-mm petri dishes. For erythroid colonies 150 000 BM cells were plated in methylcellulose medium (M3236; StemCell Technologies), supplemented with 50 ng/mL mSCF, 50 ng/mL hTPO and 5 U/mL human erythropoietin (Epo; Apoteket Farmaci) and P/S (Invitrogen). Colony numbers were scored after 12 days of culture. For total colony capacity of cultured CD34^+^ human cells, 250–300 cells were plated in 35-mm petri dishes in methylcellulose medium (M4230; StemCell Technologies) containing hSCF (25 ng/mL), GM-CSF (50 ng/mL, R&D), hIL-3 (25 ng/mL, PreproTech), hEPO (5 U/mL, Apoteket Farmaci, Lund, Sweden), and P/S (Invitrogen). Total colony number was scored after 12–14 days of culture. Cells were incubated at 37 °C in 5% CO_2_.

### Quantitative reverse transcriptase-polymerase chain reaction (qPCR)

RNA from sorted wild type cells or PEDF knockout cells was isolated (RNeasy; Qiagen) and reverse transcribed (SuperScript III, Invitrogen) in the presence of random hexamers. Quantitative PCRs (Tacman; Applied Biosystems) were performed in an ABI Prism 7700 (Applied Biosystems) according to the manufacturer’s protocol with gene-specific primers (Applied Biosystems). Each assay was performed in triplicate and the results were normalized to the housekeeping gene hypoxanthine guanine phosphoribosyl transferase (Hprt).

### Cord blood

Cord blood (CB) was obtained from Lund University Hospital, Sweden, in accordance with procedures approved by the human ethics committee at Lund University Hospital. All experiments with cord blood were carried out in accordance with the relevant guidelines and regulations from the human ethics committee at Lund University Hospital. The mothers of the babies gave informed consent by signing an informed consent form to allow the use of the CB for research purposes. For all experiments, CB samples were used within 24 hours of delivery.

### Purification of CD34^+^ cells from CB

Mononuclear cells were purified from CB using Lymphoprep (Axis-Shield, Oslo, Norway) and density gradient centrifugation according to the manufacturer’s instruction. Cells were then pooled in PBS (Invitrogen, Carlsbad, CA) with 2 mM Ethylenediaminetetraacetic acid (EDTA; Invitrogen) and 5% FCS, and washed once. The pellet was re-suspended in 300 μl PBS/EDTA/FCS and mixed with 100 μl FcR Blocking Reagent (Qiagen, Hilden, Germany). Cells were incubated with 100 μl CD34-conjugated MicroBeads (Qiagen) on shaker at 4 °C for 30 min. After washing, a MidiMACS column (Qiagen) was used to purify CD34^+^ cells, according to the manufacturer’s instructions.

### *In vitro* culture and colony assays of human CB cells

Cells were cultured in SFEM medium (Stem Cell Technologies) with P/S (Invitrogen), supplemented as indicated with hSCF (25–100 ng/ml, PreproTech), hTPO (100 ng/ml, PreproTech), Flt3L (100 ng/ml, PreproTech). CB cells were incubated at 37 °C in 5% CO_2_ with or without recombinant human PEDF (BioProducts MD, Middeltown, MD) at concentrations indicated. Cells were passaged and analyzed by FACS at indicated time points. For colony assays, cells were plated in 35-mm Petri dishes in methylcellulose medium (M4230; Stem Cell Technologies) containing hSCF (25 ng/ml), GM-CSF (50 ng/ml, R&D) hIL-3 (25 ng/ml, PreproTech) hEPO (5U/ml, Apoteket Farmaci) and P/S (Invitrogen).

### Apoptosis assay

For apoptosis assays CB cells cultured for five days were analyzed. Cells were washed twice and resuspended in staining buffer containing Annexin V-PE and 7AAD (BD Bioscience) according to the manufacturer´s protocol (BD Bioscience). Cells were incubated for 15 min and analyzed for AnnexinV binding and 7AAD incorporation.

### Statistical analysis

Data were analyzed using paired Student paired *t* test or Mann-Whitney test. *P* < .05 was considered significant.

## Electronic supplementary material

Supplementary Information
