# Supplementary material for: The stem cell regulator PEDF is dispensable for maintenance and function of hematopoietic stem cells
Source: Sci Rep. 2017 Aug 31;7:10134. doi: 10.1038/s41598-017-09452-2 (PMC5579195; doi:10.1038/s41598-017-09452-2)
Supplement: Supplementary file 1 — Supplementary Information [file 41598_2017_9452_MOESM1_ESM.pdf]

The stem cell regulator PEDF is dispensable for maintenance and function of hematopoietic stem cells

Emma Rörby[1], Matilda Billing[1], Maria Dahl[1], Sarah Warsi[1], Silja Andradottir[1], Kenichi Miharada[1], Kavitha Siva[1], Jan-Ingvar Jönsson[2], Ulrika Blank[1], Göran Karlsson[3] and Stefan Karlsson[1,*]

SUPPLEMENTARY IMFORMATION

Supplementary Figure 1. Efficient knockout of PEDF in mutant mice. (A) Expression of PEDF receptor (PEDFR) on LT-HSC (LSKCD150+CD48-), MPP (LSKCD150-CD48-) and lineage restricted progenitors (LSKCD150-CD48+) (n=6). **(B)** PEDF expression in HSCs from PEDF-deficient (KO) or wild type (WT) littermate controls. The figure shows relative mRNA levels normalized to the loading controls (n=3).

Supplementary Figure 1

A

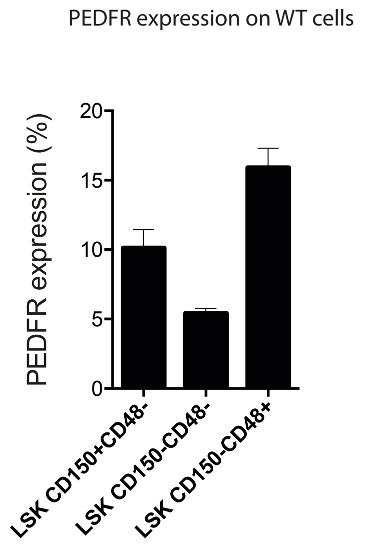

B

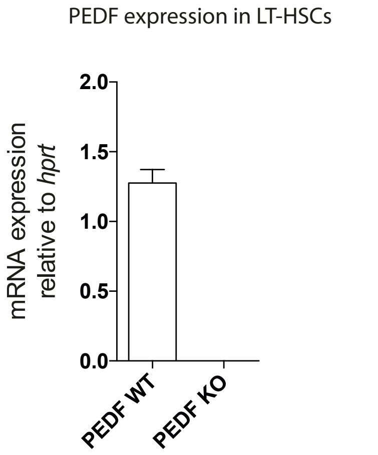

Supplementary Figure 2. PEDF expression in human CB cells. (A) Wild type cells were sorted for Lin-CD38-CD34+CD45RA-CD90+CD49f+ (HSC), Lin-CD38-CD34+CD45RA-CD90- (MPP) and Lin-CD38-CD34+CD45RA+CD90- (Myeloid Lymphoid progenitors; MLP) and PEDF mRNA expression was measured by qPCR. Line shows mean expression ± SEM (n=3).

Supplementary Figure 2

A

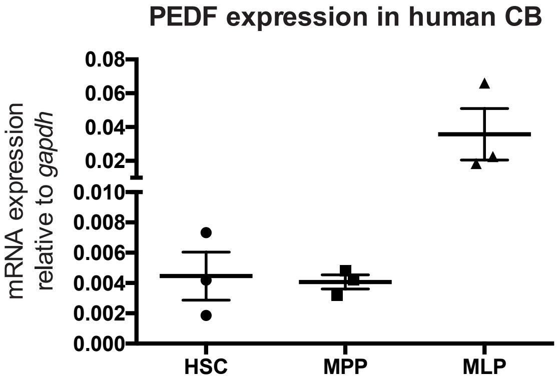

Supplementary Table 1. FACS antibodies.

Annexin-V	PE	BD Bioscience
B220	PECy5, FITC	BioLegend
CD3	PECY5, PE	BioLegend
CD4	PECy5	BioLegend
CD8	PECy6	BioLegend
CD9	FITC, PE	BioLegend
CD34	Biotin	eBioscience
CD45.1	PE, PECy7, PerCP	BioLegend
CD45.2	APC	BioLegend
CD48	FITC	BioLegend
CD150	PECy7	BioLegend
Flt3	PE	BioLegend
Gr-1	PECy5, FITC	BioLegend
Mac-1	PECy5, FITC	BioLegend
Sca-1	PacBlue, PECy7	BD Pharmingen, BioLegend
Streptavidin	Qdot605	BioLegend
PEDFR	PE	R&D Systems
Ter119	PECy5	BioLegend
human CD2	PECy5	Biolegend
human CD3	PECy5	Biolegend
human CD14	PECy5	EBioscience
human CD16	PECy5	Biolegend
human CD19	PECy5	Biolegend
human CD34	FITC	Biolegend
human CD38	APC	BioLegend
human CD45RA	APC-Cy7	Biolegend
human CD49f	PECy7	eBioscience
human CD56	PECy5	BioLegend
human CD90	PE	BioLegend
human CD123	PECy5	BioLegend
human CD235a	PECy5	BD bioscience
